# Neurovascular responses to neuronal activity during sensory development

**DOI:** 10.3389/fncel.2022.1025429

**Published:** 2022-11-11

**Authors:** Lukas Konecny, Rafid Quadir, Abel Ninan, Adrián Rodríguez-Contreras

**Affiliations:** ^1^Department of Biology, City College, The City University of New York, New York, NY, United States; ^2^The Roxelyn and Richard Pepper Department of Communication Sciences and Disorders, School of Communication, Northwestern University, Evanston, IL, United States

**Keywords:** hypoxia, onset of hearing, neurovascular unit, astrocytes, hemodynamics

## Abstract

Understanding the development of intercellular communication in sensory regions is relevant to elucidate mechanisms of physiological and pathological responses to oxygen shortage in the newborn brain. Decades of studies in laboratory rodents show that neuronal activity impacts sensory maturation during two periods of postnatal development distinguished by the maturation of accessory structures at the sensory periphery. During the first of these developmental periods, angiogenesis is modulated by neuronal activity, and physiological levels of neuronal activity cause local tissue hypoxic events. This correlation suggests that neuronal activity is upstream of the production of angiogenic factors, a process that is mediated by intermittent hypoxia caused by neuronal oxygen consumption. In this perspective article we address three theoretical implications based on this hypothesis: first, that spontaneous activity of sensory neurons has properties that favor the generation of intermittent tissue hypoxia in neonate rodents; second, that intermittent hypoxia promotes the expression of hypoxia inducible transcription factors (HIFs) in sensory neurons and astrocytes; and third, that activity-dependent production of angiogenic factors is involved in pathological oxygen contexts.

## Introduction

**Figure 1:**
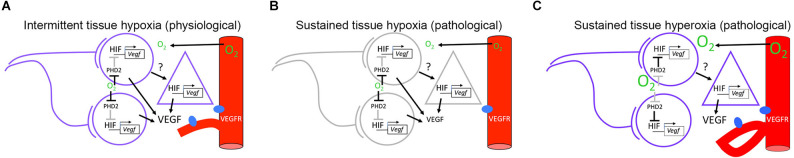
Hypothetical model for neuronal activity modulation of angiogenesis in neonate rodents. **(A)** Under environmental normoxic conditions, co-active neurons contribute to generate an intermittent hypoxic environment that favors proangiogenic signaling through the HIF/VEGF pathway. Neuron to astrocyte communication may contribute to VEGF production through unidentified signals. **(B)** Under environmental hypoxic conditions, a sustained hypoxic environment is imposed in the brain parenchyma. Sustained hypoxia inhibits electrical activity in neurons and potentially, signaling from neurons to astrocytes. Despite favorable conditions for HIF/VEGF signaling, VEGF levels are reduced resulting in a decrease in the angiogenic response. **(C)** Under environmental hyperoxic conditions, a sustained hyperoxic environment is imposed on the brain parenchyma. Sustained hyperoxia is favorable for neuronal activity but not for HIF/VEGF signaling in neurons. Altered VEGF release and aberrant angiogenic responses are hypothesized to originate from abnormal neuron to astrocyte signaling. Circles represent neurons. Triangles represent astrocytes. Red cylinders represent blood vessels expressing VEGF receptors (VEGFR), shown in blue. Active cells and pathways are shown in purple and black, respectively. Inactive cells and pathways are shown in gray. HIF/VEGF signaling pathway has been adapted from Leu et al. (2019). HIF, hypoxia inducible transcription factor; VEGF, vascular endothelial growth factor.

Perinatal damage to the developing brain is a major cause of death and permanent neurodevelopmental disability in the world, with oxygen shortage being an important factor that affects preterm and term neonates (Watchel et al., 2019). For example, asphyxia and hypoxic-ischemic insult are associated with hearing loss and poor speech development in humans (Pham, 2017), and with auditory brainstem processing deficits in rodent models (Hall, 1964; Kaga et al., 1996; Rehn et al., 2002; Strata et al., 2005, 2010; Jiang et al., 2009). These observations indicate that part of the pathology derived from altered oxygen supply in the neonate brain involves sensory neurons, but its effect on other cell types remains relatively unexplored. Intriguingly, recent studies showed that local tissue hypoxic events are generated in response to physiological levels of neuronal activity in the somatosensory cortex of neonate rodents (Kozberg et al., 2016), and that this occurs contemporary with a period in which angiogenesis is modulated by neuronal activity (Lacoste et al., 2014; Whiteus et al., 2014). This correlation suggests that neuronal activity is upstream of the production of angiogenic factors, a process that is mediated by intermittent hypoxia caused by neuronal oxygen consumption. In this perspective article we address three theoretical implications based on this hypothesis: first, that spontaneous activity of sensory neurons has properties that favor the generation of intermittent tissue hypoxia in neonate rodents; second, that intermittent hypoxia promotes the expression of hypoxia inducible transcription factors (HIFs) in sensory neurons and astrocytes; and third, that activity-dependent production of angiogenic factors is involved in pathological oxygen contexts (Figure 1).

### Properties of spontaneous activity of sensory neurons that favor the generation of intermittent hypoxia in neonate rodents

In rodents, developmental changes in neuronal activity of sensory neurons are observed during two periods of postnatal development. In the first period, spontaneous bursts of action potentials that originate endogenously in sensory organs propagate to the brain through neuronal connections that are refined by activity-dependent and genetically encoded mechanisms. During the second period, development of accessory structures at the sensory periphery marks the onset of sensation and initiates critical periods where synaptic and intrinsic neuronal properties continue to recalibrate in response to activity changes driven by stimuli from the environment (Knudsen et al., 2000; Wang and Bergles, 2015; Seabrook et al., 2017; Cisneros-Franco et al., 2020; Rubio, 2020). For example, between birth (postnatal day 0, P0) and P12 cochlear inner hair cells in the auditory system fire spontaneous bursts of calcium action potentials that drive activity-dependent synaptic refinement and maturation of intrinsic properties in central auditory neurons (Tritsch et al., 2007, 2010; Kandler et al., 2009; Johnson et al., 2011, 2012; Clause et al., 2014; Sendin et al., 2014; Di Guilmi et al., 2019). In turn, around P10 in mice and P12 in rats, formation of the ear canal and clearance of mesenchyme from the middle ear cavity mark the beginning of experience-dependent plasticity in the auditory system (Sanes and Bao, 2009; de Villers-Sidani and Merzenich, 2011; Adise et al., 2014; Anthwal and Thompson, 2015).

Although different cellular mechanisms are involved in the generation of spontaneous neuronal activity in different sensory organs before P12, electrophysiology recordings showed that action potential bursts are the predominant firing pattern in individual visual and auditory neurons of neonate rodents (Mooney et al., 1996; Sonntag et al., 2009; Tritsch et al., 2010). It is important to note that during burst activity the firing rates of individual auditory neurons vary across three orders of magnitude. This means that although action potential bursts occur very infrequently (30–100 mHz), maximal firing-rates within bursts can reach between 10 Hz and up to 100 Hz for bursts that last from 2 to 12 s (Tritsch et al., 2010). In addition, multi-electrode recordings in the auditory brainstem of neonate rats showed evidence that the levels of ensemble neuronal activity increase from birth until hearing onset, reaching a maximum at P9 (Di Guilmi and Rodríguez-Contreras, 2021).

Classic studies in the mammalian visual system have shown that optical recordings of neuronal activity can provide detailed information about the spatiotemporal properties of spontaneous ensemble neuronal activity, complementing the information obtained with electrophysiological methods (Meister et al., 1991; Wong et al., 1993, 1995). In recent studies, transgenic mice that express genetically encoded calcium indicators in neurons were used to show that ensembles of sensory midbrain and cortical neurons exhibit highly synchronized activity with different modes of propagation. In the visual system, ensemble neuronal activity propagates through waves of irregular trajectories that resemble the waves of neuronal activity generated in the retina (Wong et al., 1995; Ackman et al., 2012). In the auditory system, ensemble neuronal activity is spatially restricted, and resembles the spatiotemporal activation of hair cells in the cochlea (Tritsch and Bergles, 2010; Babola et al., 2018). Additional calcium imaging studies in mouse auditory midbrain showed that spontaneous ensemble neuronal activity becomes more frequent and spatially refined between birth and P12 (Wang et al., 2021). Despite the different modes of propagation, spontaneous ensemble neuronal activity is stochastic, and highly variable in amplitude and duration, which implies participation of different numbers of cells during each activation event (Ackman et al., 2012; Babola et al., 2018). Altogether, these results raise new questions about the significance of the spatiotemporal features of spontaneous neuronal activity during postnatal development.

The significance of the spatiotemporal changes of neuronal activity before P12 has been addressed in the context of synaptic development, where neurons that fire together wire together (Butts et al., 2007). However, recent studies motivated to characterize the development of neurovascular responses in neonates have provided novel and intriguing results for alternative lines of investigation. Using genetic targeting of calcium indicators in neurons, Kozberg and colleagues showed that sensory stimulation reliably activates localized neuronal ensembles in the somatosensory cortex but fails to cause a significant increase in blood flow at ages P7–P8. In contrast, at ages P12–P15 and adulthood, sensory neuron activation leads to robust increases in blood flow due to neurovascular coupling (Kozberg et al., 2016). Furthermore, the authors obtained simultaneous optical measurements of neuronal activity and local changes in oxygenated hemoglobin. This approach demonstrated the existence of local hypoxic events in P7–P8 mice, particularly during long periods of ensemble neuronal activity, whether they were initiated by sensory stimulation or occurred spontaneously (Kozberg et al., 2016; reviewed in Kozberg and Hillman, 2016).

Altogether, the predominance of burst firing in spontaneously active sensory neurons, the developmental increase in the number of co-active cells in ensembles, and the recent discovery that blood flow does not increase in response to neuronal activity in neonates before P12, imply that sensory neurons that fire together contribute to generate a tissue environment characterized by intermittent hypoxia, despite the fact that neonates breathe in a normoxic environment (Figure 1A).

### Intermittent hypoxia favors the expression of hypoxia inducible factors (HIFs) in sensory neurons and astrocytes

Vascular development and homeostasis are partly regulated by vascular endothelial growth factor (VEGF), a secreted polypeptide that is produced by tissues in response to hypoxia. VEGF activates receptors on vascular endothelial cells that promote their survival, proliferation, and migration toward the VEGF source (Chung and Ferrara, 2011; Leu et al., 2019). Because transcription of the *Vegf* gene is controlled by HIFs, α–β heterodimeric transcription factors that are stabilized by tissue hypoxia (Semenza, 2014), different studies have used genetic manipulations of this signaling pathway in the retina and the brain of neonate mice to determine its effects on vascular development.

In the retina, the three vascular layers: the external, the deep and the intermediate plexuses, begin to form at birth, at P7 and at P11, respectively. Genetic activation or suppression of HIF/VEGF signaling in retinal horizontal or amacrine cells results in inversely modulated intermediate plexus vascularization (Usui et al., 2015). When genetic suppression of HIF/VEGF in neurons was compared to genetic suppression of HIF/VEGF in astrocytes, the results indicated that neurons and astrocytes are sources of VEGF that affect angiogenesis of the intermediate plexus. Interesting to us, broad suppression of VEGF also affected astrocyte migration, while suppression of VEGF only in astrocytes affected endothelial cell migration. This suggests that paracrine and autocrine VEGF have different effects in the proliferation and migration of retinal endothelial cells and the migration of astrocytes, respectively (Rattner et al., 2019).

Tissue hypoxia stabilizes the expression of HIFs by inhibiting the HIF suppressing action of prolyl-4-hydroxylase domain (PHD) enzymes (Rey and Semenza, 2010; Leu et al., 2019). PHD2 is the most abundant isoform in neurons of the mouse brain (Rabie et al., 2011; Segura et al., 2016), and has been identified to be the critical oxygen sensor setting the low steady-state levels of HIFs in normoxic conditions (Berra et al., 2003). Nasyrov and colleagues generated *Phd2*-deficient and *Hif1a/Hif2a*-deficient mice to demonstrate that perinatal activation or suppression of HIF signaling in excitatory forebrain neurons inversely modulates angiogenesis at age P7 but not at birth (Nasyrov et al., 2019). When *Phd2/Hif1a/Hif2a*-deficient animals were generated and analyzed, the authors found that in addition to HIF1a stabilization, VEGF mRNA levels were increased not only in neurons but surprisingly also in astrocytes. Although the molecular mechanism was not identified, this result underscores the relevance of signaling between neurons and astrocytes (Nasyrov et al., 2019).

Altogether, the results of neuron and astrocyte targeted genetic manipulations in neonate mice show evidence that these cells express HIFs at the developmental period when spontaneous activity of sensory neurons is at its peak. Furthermore, these studies also show that specific types of neurons are a major source of VEGF for local angiogenesis, and that astrocytes can produce VEGF in response to neuronal signaling. Next, we address studies that demonstrate a relationship between neuronal activity and postnatal angiogenesis in physiological and pathological contexts.

### Activity-dependent production of angiogenic factors is involved in pathological oxygen contexts

In the retina, the development of the external and deep vascular plexuses overlaps with a period of cholinergic neural activity driven by starburst amacrine cells (SAC), the only cholinergic neurons during the P0–P10 developmental stage (Seabrook et al., 2017). By using a combination of SAC ablation, pharmacological blockade of cholinergic activity, and chemogenetic inhibition of SAC activity from P3 to P9, Weiner and colleagues demonstrated that SAC activity is involved in vascularization of the deep retinal layer, but not the external layer by a decrease in VEGF (Weiner et al., 2019). Furthermore, in this study the authors showed that inhibiting cholinergic activity also reduced the vascular defects in a mouse model of oxygen-induced retinopathy (Weiner et al., 2019). The results of this study show that neuronal activity lies upstream of VEGF and highlight the important role of this signaling pathway in developmental and pathological angiogenesis (Kurihara et al., 2014; Weiner et al., 2019).

## Discussion

In this perspective article, we hypothesized that neuronal activity is upstream of the production of angiogenic factors, a process that is mediated by intermittent hypoxia caused by neuronal oxygen consumption. Next, we discuss alternative ideas that support or argue against this hypothesis, and propose potential areas for future research.

Although we have argued that the spontaneous electrical activity of sensory neurons has properties that favor the generation of intermittent tissue hypoxia in postnatal mice, it is important to acknowledge that synaptic neurotransmission between neurons may play a substantive role in linking neuronal activity to responses from other cells of the neurovascular unit. This interpretation is supported by the studies of cholinergic SAC in the retina, which are cells that do not fire action potentials, and nevertheless play integral roles in synaptic communication with other SAC and with retinal ganglion cells (Seabrook et al., 2017; Weiner et al., 2019). Others have also proposed that the dependence of angiogenesis on neuronal activity may be restricted to specific circuits and CNS regions at different periods in development (Weiner et al., 2019).

We have also argued that neuronal activity promotes the expression of HIFs in sensory neurons and astrocytes by creating an environment of intermittent hypoxia. Others have proposed that developmental angiogenesis is dependent on additional energy consuming processes such as myelination (Yuen et al., 2014). Without a quantification of oxygen consumption by different cellular processes, it will be difficult to argue in favor of a general role of neuronal activity in different regions of the brain. It is clear however, that VEGF is a main factor regulating angiogenesis in the retina and the brain of neonates, and that VEGF is produced by specific types of neurons and by astrocytes (Usui et al., 2015; Nasyrov et al., 2019; Weiner et al., 2019). Additional signaling roles for oligodendrocytes, microglia, and mural cells cannot be excluded (Yuen et al., 2014; Biswas et al., 2020; Huang, 2020).

As participants of the tripartite synapse, astrocytes communicate with neurons and regulate neurotransmitter processes (Araque et al., 2014; Durque and Araque, 2019). Thus it is possible that synaptic communication could confer the hypoxic signal to neighboring cells through spillover of co-transmitters such as ATP, acetylcholine or to-be-identified transmitters released from neurons to astrocytes (Nasyrov et al., 2019; Weiner et al., 2019). Relevant examples of communication between sensory cells and glia exist in the developing auditory system. For example, glia-like supporting cells in the cochlea have been identified as key initiators of inner hair cell depolarization and calcium spiking *via* the extracellular release of potassium ions modulated by purinergic signaling (Tritsch et al., 2007; Babola et al., 2020). A recent calcium imaging study in the mouse auditory midbrain also showed that spontaneous bouts of ensemble neuronal activity triggered the co-activation of astrocytes by inducing calcium release from intracellular stores through the activation of metabotropic glutamate receptors, mGluR5, and mGluR3 (Kellner et al., 2021). The significance of these forms of co-activation between sensory cells and glial cells for HIF/VEGF signaling and postnatal angiogenesis remain to be established.

Lastly, we considered that activity-dependent production of angiogenic factors is altered in pathological oxygen contexts. The severity of brain damage caused by altered oxygen levels may be a function of the developmental stage, the direction of oxygen change, and the spatiotemporal spread of the effect. We believe that distinction between intermittent physiological and sustained pathological oxygen changes is important. Figures 1B,C shows a hypothetical view of pathological contexts. First, because neurons have a high energy turnover and rely on aerobic metabolism, we can expect that an environmental decrease in oxygenation would lead to a sustained decrease in tissue oxygen levels, with subsequent inhibition of neuronal activity (Figure 1B, Lujan et al., 2021). If VEGF production was dependent on neuronal activity, this would lead to hypovascularization, although compensatory mechanisms could be observed. In contrast, neuronal activity may produce aberrant new vessels in the presence of increased oxygen levels (Figure 1C, Weiner et al., 2019). In addition, it is important to acknowledge that changes in oxygen levels may be accompanied by other factors such as aglycemia and inflammation during ischemic-hypoxic contexts, which could have different effects in gray matter compared to white matter regions (Tekkök et al., 2003).

Despite the above considerations, it remains to be determined how the effects of acute oxygen changes translate into long-term deficiencies in sensory processing, motor function, and cognition. Functional magnetic resonance imaging (fMRI) studies in the somatosensory system and the visual system of rats showed gradual increases in the magnitude of the blood oxygen level-dependent (BOLD) signal in response to sensory stimulation between P13 and adulthood (Colonnese et al., 2008; Chan et al., 2010), which were in agreement with earlier studies of sensory plasticity, where changes in regional manganese-enhanced MRI activity were detected in the auditory midbrain of mice reared in control and experimental sound conditions between P13 and P19 (Yu et al., 2007). Could alterations in activity-dependent angiogenesis during early postnatal development be related to long-term defects in neurovascular coupling? Although the mechanisms that trigger neurovascular coupling are under intense investigation (Kaplan et al., 2020), future work can focus in a detailed examination of the onset of neurovascular coupling and its relationship to recent angiogenic activity in relevant auditory and voice control regions of animal models, or in human subjects with non-invasive approaches.

## Data Availability Statement

The original contributions presented in the study are included in the article, further inquiries can be directed to the corresponding author.

## Author Contributions

AR-C conceived the idea and wrote the final version of the manuscript with input from all the authors. LK, RQ, AN, and AR-C discussed and drafted the manuscript. RQ and AR-C generated figures. All authors contributed to the article and approved the submitted version.

## Funding

This work was supported by startup funds from Northwestern University to AR-C.
